# Meum and Teum

**Published:** 1843-04

**Authors:** 


### "MEUM AND TEUM."

Under the above head (which may or may not contain a typographical error) the *Medical News*, which seems to aspire to the respectable office of *tender* to the *American Journal of the Medical Sciences*, holds the following language:

"*Meum and Teum.*—We observe that the editors of the *Western Journal of Med. and Surgery* have done the editor of the *American Journal of the Medical Sciences* the honor to estimate several of the articles of his quarterly summary of sufficient value to be transferred to the pages of their Journal. They have considered the compliment of making use of his labours, however, sufficient, without thinking it necessary to make the acknowledgment which bare justice requires in such a case."

We freely acknowledge our indebtedness to the American Journal, as well as to other eastern periodicals, for foreign news, and the pages of this Journal will show that it has been our custom to make the usual "acknowledgment"—to a greater extent perhaps than some of our contemporaries, equally indebted as ourselves. There is scarcely a number of it that does not contain articles credited to the American Journal; and if such credit has been at any time improperly omitted, the editor of that Journal will, we are sure, do *us* the "justice" to believe that it was done inadvertently. We shall endeavor to avoid such omission in future.

The paragraph we have quoted strikes us as being tolerably *pert*, to come from one who, so far as we can see, has no interest whatever in the matter. It would be well for its author, perhaps, to polish both his manners and his Latin, ere he takes us to task again. We would also recommend him, in view of his disinterestedness on this occasion, *posthac in res suas diligenter incumbere.* We have couched this last in Latin, because, as he seems to have a fondness for the classics, it may thereby more readily attract his notice. Attention to these two suggestions may possibly keep him out of trouble. C.

